# Death of Dr. Samuel Hogg

**Published:** 1842-08

**Authors:** 


### DEATH OF DR. SAMUEL HOGG.

Died, on the 28th of May, at his residence in Rutherford County, Tennessee, Dr. Samuel Hogg, one of the oldest and most eminent physicians of that State. The disease which terminated the life of this estimable man was pulmonary consumption, of which he had been the subject for a number of years. At one time in the summer of 1838 it was feared he would sink under the disease, but by the autumn he had so far recovered as to be able to return to Natchez, where he then resided, and resume the practice of physic. With the return of warm weather, however, his malady grew more alarming, and he was obliged to fly again to the mineral springs of Tennessee, where the recuperative process went on so kindly, that he was found the next winter, in Nashville, laboring in his profession, and the next spring taking an active part in the business of the Medical Society of the State. At the meeting of the Society, in May of the following year, he delivered the annual address, as President, which was the last public effort of his life. The summer was passed, as usual, at the watering places in the mountains of Tennessee, and in the winter, his health having sensibly declined, he exchanged his house in Nashville for a farm in the country, to which he retired in March, to sink soon into the grave.

Dr. Hogg was a native of North Carolina, and was born on the 18th of April, 1783, of respectable parents, his father having served his country as Major in the war which achieved our independence, and his mother, who was a widow at the time of her marriage with Major Hogg, having been equally remarkable for her good sense, spirit, and devotion to liberty. It is asserted, on authority believed to be indisputable, that it was this lady, then the widow Webb, who made the reply to Tarlton, memorable for its withering sarcasm, that "he *might* have enjoyed the pleasure of seeing Col. Washington, of which he then seemed so desirous, *if he had only taken time to look behind him at the battle of the Cow Pens.*" Dr. Hogg inherited a large share of his mother's courage and mental activity, and as he lost his father very young he was probably indebted chiefly to her for the early training of his mind. He received instruction, for some time, at a high school in Caswell County, where he acquired some knowledge of the Latin tongue, and became sufficiently versed in the various branches of English education to take charge of a school for boys, which he taught for a short time. Having chosen medicine for a profession, he engaged in the study of it under the direction of Dr. John Hare, and with such aids as his preceptor could afford him, entered upon the practice after the course of reading customary at that day. His first settlement was at Gallatin, in Tennessee, but he was induced, after a few months, to remove to Lebanon, in a neighboring county, where there was less competition. At this place the late war of this country with England found him, and obeying the impulses of his nature, as well as the dictates of duty, he accepted the appointment of Surgeon to the troops that descended the Mississippi to Natchez in 1813.

He returned from this service with an experience greatly enlarged, and with a high reputation for professional boldness, discrimination, and skill. The winter following, in a similar capacity, he accompanied the Tennessee militia on the campaign against the Creek Indians. We have before us an interesting memorial of this campaign, in a brief notice written by him of the measles which broke out among the soldiers in January and prevailed for many weeks as an afflicting epidemic. This account is short, but describes with clearness the peculiar features of the epidemic, and contains indications of that decision and energy in practice for which Dr. Hogg was especially distinguished. In the winter of 1814 he attended the troops to New Orleans, and was present at the memorable battle of the 8th of January. He was about rising in the morning when the first cannon announced the commencement of the conflict, and it is an interesting incident in his life, that the servant who was handing him water to wash was killed by a cannon-ball which carried away his head, spattering his brains in the Doctor's face.

During the encampment at New Orleans, influenza, in its most violent form, broke out among the troops, and it was in his ceaseless attendance on his afflicted countrymen, bringing an attack of the epidemic on himself, that, according to his own belief, he laid the foundation of that disease to which at length he fell a victim. This complaint appearing in the extreme north-east of the United States, and spreading over the country to the south-west, and termed, variously, cold plague, influenza, pneumonia typhoides, &c., is well remembered by the profession as, perhaps, the most fatal epidemic that has visited the country since its first settlement. Whole families, consisting, in some instances, of fifteen or twenty members, were cut off, and localities the most healthful were visited by it with a mortality not exceeded in cities subject to the visitations of yellow fever or the plague. The plans of treatment pursued in it were as diverse as the localities where it appeared were distant from each other—in some, the practice being to support the sick by cordial remedies; while in others nothing was found to succeed but the most active antiphlogistic treatment. Among the advocates for the latter practice Dr. Hogg was one of the most enthusiastic and successful. One of the sayings which we remember as reported of him during the sway of the epidemic was, "that *his patients should not die of the disease—he would bleed them to death first!*" By universal consent, no practitioner in the army, or in his neighborhood, where he encountered the disease the winter subsequent to his return home, surpassed him in the number of his cures. In truth, his fame as a practitioner rose, in consequence of his success in this epidemic, to a height which has not been exceeded by that of any physician in that State.

To his professional accomplishments Dr. Hogg had the good fortune to add all those qualities of the man which most captivate and win men's hearts. He was brave, confiding, social, liberal to a fault, of conversational abilities the readiest and most agreeable, and with incidents in his own eventful life upon which to exert them to the highest advantage. Soon after his services in the army he was induced by the wishes of his countrymen to become a candidate for civic honors, and once he was chosen the Representative of his county in the Legislature of the State, and once was elected to Congress. It was while serving his country at Washington, in 1819, that the University of Maryland conferred on him the honorary degree of *Doctor of Medicine*, which was followed, some years afterwards, by a similar acknowledgment of his high professional character from Transylvania University.

Dr. Hogg's reputation was now in its zenith, and as he was of an active, inquisitive mind, that acquired with uncommon readiness and ease whatever circulated in any of the channels of the profession, he had only to direct his affairs with prudence, to render his circumstances in life as independent as he could have desired. At this time his services were in perpetual requisition. His connexion with the army during so many campaigns had extended a knowledge of his skill all over the State, and he was consulted by the sick from every portion of it. He almost lived on horse-back, and performed for several years an amount of professional service which has seldom been surpassed by the practitioner of medicine. But labors so arduous and unmitigated began to produce their natural effects upon his health, and in order to lighten them he removed to Nashville, in 1828, where he secured at once an amount of business proportionate to his high reputation. But, having been unfortunate in his connexion with a drug store, he was tempted by the representations of friends to try his fortunes in a field promising still higher pecuniary rewards, and accordingly, in 1836, he settled in Natchez. Here, as before in Nashville, his business became at once large and profitable, and, could his health have been prolonged, it is probable he would have amassed riches. But unfortunately that failed him after a few months, and he was compelled to give up his cherished schemes, only to return to them for a little while once more, and then abandon them forever. In 1838, an invalid ourself, we passed him at Tyree's Springs, where it was apprehended he was about to finish his course. It was during his illness that season that he professed religion, and was received into the Baptist church, in the faith of which he died. We had the satisfaction of seeing him promoted to the first office in the Medical Society of the State which he had honored by his services, at its meeting in May, 1840, and in 1841 listened with mournful interest to his address as the retiring President, assured by his emaciated frame and feeble voice, that we should hear him from that place no more. When next we saw him he was on his death-bed. A year had passed away, and in that time he had lost a third son, an amiable young man, the second who had prepared himself for the profession of the father. His earthly hopes had all faded, and he spoke of his dissolution as just at hand; but he spoke of it with the calm resignation of a Christian. In three weeks the end, which had been threatened long, came, and he died without a struggle. He was buried at Nashville, a large concourse of citizens attending his remains to the grave. Resolutions respectful to his memory were passed by the physicians of Murfreesborough and Rutherford County, by one of which a memoir of his life and character is requested to be read at the next annual meeting of the State Medical Society, of which he was one of the founders and most active and zealous members.

One of the medical pioneers of Tennessee, Dr. Hogg was too ambitious and too proud a man to lag behind those whose superior opportunities had enabled them to store their minds with the last improvements in the profession. He was, therefore, a reading man. He kept up with the discoveries in medicine, and with the changes in medical doctrine. He loved medical speculation, and spake and wrote fluently concerning his favorite theories, which, if never profound, were generally plausible and ingenious. He possessed a large share of energy and enthusiasm, which he carried into his profession, and these qualities gave him confidence and boldness as a practitioner, and, added to his powers of conversation, rendered him a fascinating teacher. If the character of his intellect was rather showy than solid, it will be conceded that few men, with as limited advantages of early professional learning, have maintained a more undisputed empire in their profession, than Dr. Hogg long exercised in his comparatively limited sphere. As a writer, he was not wholly unknown to the profession; but he was too sensible of the defects of his early education, to be a willing contributor to the press. As a member of society he was greatly beloved, his warm and benevolent heart attaching men to him by the strongest ties. It was late in life that he united to the generous qualities of the man the high and ennobling graces of the Christian; but it is the richest of all the sources of consolation left to his friends—and above all, to his bereaved family—that he was supported under the sufferings of his last lingering illness by the Christian's hopes. Y.

